# Leishmania and Leishmaniasis Research: The Past 50 Years and the Future

**DOI:** 10.3390/pathogens12060776

**Published:** 2023-05-30

**Authors:** Kwang Poo Chang

**Affiliations:** Department of Microbiology/Immunology, Center for Cancer Cell Biology, Immunology and Infection, Chicago Medical School, Rosalind Franklin University of Medicine and Science, North Chicago, IL 60064, USA; kwangpoo.chang@rosalindfranklin.edu

Leishmaniasis is a widespread disease among impoverished populations with an annual incidence of up to 1 million according to the WHO [1]. The disease is transmitted by the blood-feeding females of sand flies in most endemic areas and by midges in several locations. The existence of reservoir animals, such as dogs and rodents, complicates the transmission of this disease in many endemic areas. Environmental landscapes vary greatly from one endemic site to another; thus, investigations of global epidemiology are difficult and incomplete. The progression of the disease follows a chronic course in three different forms, depending on the *Leishmania* species and their interactions with the hosts. Visceral leishmaniasis, if not treated, is often fatal. This disease still plagues East Africa, South Asia, South America and many other scattered sites of low endemicity. Cutaneous and mucocutaneous leishmaniasis are non-fatal but can become protracted in a non-healing state, resulting in facial disfigurements in the latter. These cutaneous diseases remain common in Latin America, the Middle East, Central and Western Asia, and parts of Africa. Progress has been made in diagnosing and treating the disease, but antiquated methodologies and toxic drugs are still in use. The improvement of health conditions via government policy and economic development reduces the incidence of leishmaniasis. These measures have contributed to the success of programs designed specifically to eliminate visceral leishmaniasis in the major endemic sites of China and India.

This Special Issue, Leishmania and Leishmaniasis, includes articles of diverse disciplinary areas, ranging from basic science to clinico-epidemiological investigation. The articles in basic science include molecular genetics, cellular and molecular biology, and immunology to address the issues of Leishmania’s unique features, host–parasite interactions in infection and immunity, drug discovery and vaccine development. Articles in clinical science and field work are devoted to disease pathogenesis, co-infection; clinical trials of drugs; and vector biology and epidemiology. The coverall approach for this volume is in line with the idea of One Medicine, One Health, One World [2], that is designed to promote communication among the divergent disciplinary areas related to leishmaniasis as a disease entity. The push for this approach began in the early 1970s when the public and private sectors joined forces to provide significant support for laboratory research on parasites and field work on tropical diseases to tackle problems such as the global spread of leishmaniasis. Considerable progress has been made in the last 50 years, beginning with effective cultivation of Leishmania in the lab and the establishment of clinics dedicated to leishmaniasis. Advances in Leishmania laboratory studies have closely followed developments in the basic sciences, from biochemistry to cellular/molecular biology to omics analyses. Today, field stations, clinics and hospitals have been established specifically to deal with leishmaniasis in a number of endemic sites in different parts of the world. The articles compiled in this volume are representative of the fruitful outputs from these advances.

Past accomplishments in Leishmania and leishmaniasis research have undoubtedly provided a sound foundation for future investigations. In the short term, further progress is expected to build on the current approaches. In the long term, it is necessary to adopt new approaches, which offer high hopes for accelerated achievements in bench-side research to develop products for bedside applications. The greatest achievements in basic and clinical sciences will certainly be the utility of data science technology assisted by artificial intelligence coupled with machine learning/deep learning programs [3,4]. Basic biological and biomedical research has already begun to deploy these new approaches. Information is urgently needed for infectious diseases such as leishmaniasis about how infection is initiated in humans and the subsequent host–parasite interactions that lead to the manifestation of clinical symptoms. A potential tool for such investigations may be 3D human organoid cultures [5,6], with which vascular blood flow can be provided to supply the immune cells necessary for the study of infection and immunity [7,8]. The individualized organoids of such designs have the potential to facilitate precision or personalized medicine, which is predicted to be essential for future healthcare [9] by, for example, supplementing or replacing population-based clinical trials of drugs and vaccines for infectious diseases. These approaches are expected to become available in the future for the study of neglected diseases such as leishmaniasis.
